# Load Monitoring Practice in European Elite Football and the Impact of Club Culture and Financial Resources

**DOI:** 10.3389/fspor.2021.679824

**Published:** 2021-05-20

**Authors:** Kobe C. Houtmeyers, Jos Vanrenterghem, Arne Jaspers, Ludwig Ruf, Michel S. Brink, Werner F. Helsen

**Affiliations:** ^1^Faculty of Movement & Rehabilitation Sciences, Catholic University (KU) Leuven, Leuven, Belgium; ^2^Institute of Sports and Preventive Medicine, Saarland University, Saarbrücken, Germany; ^3^Center for Human Movement Sciences, University of Groningen, University Medical Center, Groningen, Netherlands

**Keywords:** soccer, physical training, team sport, technology, sports science, analysis

## Abstract

Load monitoring is considered important to manage the physical training process in team sports such as Association Football. Previous studies have described the load monitoring practices of elite English football clubs and clubs with an established sports-science department. An examination of a broader international sample is currently not available. In addition, previous research has suggested factors that may improve the implementation of load monitoring practices, such as a strong club belief on the benefit of evidence-based practice (EBP) and high club financial resources. However, no study has examined yet the actual impact of these factors on the monitoring practices. Therefore, this study aims (1) to provide an overview of load monitoring practices in European elite football and (2) to provide insight into the differences in implementation between clubs by examining the impact of the club beliefs on the benefit of EBP and the club financial resources. An online survey, consisting of multiple choice and Likert scale questions, was distributed among sports-science and sports-medicine staff (*n* = 99, 50% response rate). Information was asked about the types of data collected, collection purposes, analysis methods, and staff involvement. The results indicated that external load data (e.g., global navigation satellite system, accelerometer…) was collected the most whilst respondents also indicated to collect internal load (e.g., heart rate, rating of perceived exertion…) and training outcome data (e.g., aerobic fitness, neuromuscular fatigue…) for multiple purposes. Considerable diversity in data analysis was observed suggesting that analysis is often limited to reporting the gathered data. Sports-science staff were responsible for data collection and analysis. Other staff were involved in data discussion to share decision-making. These practices were positively impacted by a stronger club belief on the benefit of EBP and greater financial resources. Creating an organizational culture, characterized by a strong belief on the benefit of EBP, is important to increase the impact of load monitoring. However, the actual potential may still be largely determined by financial resources. High-level clubs could therefore play a leading role in generating and sharing knowledge to improve training practices and player health.

## Introduction

Load monitoring currently receives much attention in elite team sports such as Association Football. It is considered the systematic quantification, analysis and management of the training load, with the aim of achieving positive training outcomes such as an improved fitness and reduced fatigue (Impellizzeri et al., 2019). While external load is defined as the physical work prescribed in the training plan, internal load reflects the psychological, physiological and mechanical stress experienced by players during the training (Vanrenterghem et al., 2017; Impellizzeri et al., 2019). The internal load for a given external load varies between or within players depending on the player characteristics (e.g., endurance) and the context of the training (e.g., temperature). The combined monitoring of external and internal load in relation to training outcomes provides insight about players' responses to training and can be used to plan future training.

Previous studies have described the load monitoring practices in elite English football (Weston, 2018) and in an international convenience sample of clubs with an established sports-science department (Akenhead and Nassis, 2016). Both studies reported an extensive implementation of load monitoring practices. However, although Weston (2018) reported that staff members generally perceive load monitoring as beneficial, a considerable diversity in response was observed to the question if load monitoring was used positively or negatively. Likewise, Akenhead and Nassis (2016) reported that staff members perceived the actual effectiveness, based on experience, as lower than the expected effectiveness, based on theoretical scientific concepts. These observations indicate that the process of implementation is not always straightforward and emphasize the importance of converting data into actionable insights (Gamble et al., 2020). Both studies provide useful information on the current implementation of monitoring practices. However, the results of these studies must be interpreted in relation to the population that was surveyed. The implementation of monitoring practices in elite football may be overestimated when the current findings are generalized to clubs without a sports-science department or to clubs belonging to other national federations than the high-level English federation. Therefore, a description of load monitoring practices in a broader international sample is required to better understand the current implementation of monitoring practices in elite football. In addition, such an examination may provide more insight into the difference in implementation between clubs, which may depend on cultural and financial factors (Finch, 2006; Bishop, 2008).

The organizational culture and financial resources are known to be important factors in translating research and knowledge into practice in different settings such as health services, business organizations and sport-related settings such as injury prevention (Argote and Ingram, 2000; Grimshaw et al., 2012; McKay and Verhagen, 2016; Buckthorpe et al., 2018). In football, factors related to club culture and resources were also suggested to influence the implementation of load monitoring practices, but have not been studied yet (Akenhead and Nassis, 2016; Weston, 2018; Fullagar et al., 2019). A club culture that is characterized by a strong belief on the benefit of evidence-based practice (EBP), defined as the integration of staff members' expertise, athlete values and research evidence in decision-making, may strengthen staff members' commitment to load monitoring (Fullagar et al., 2019). Availability of financial resources may further facilitate the implementation by allowing the purchase of measurement technology or the recruitment of specialist staff members. Although previous studies surveying staff members' perceptions suggested an influence of these factors, no study has yet examined the actual impact of the club belief on the benefit of EBP and the club financial resources on the implementation of load monitoring practices.

Therefore, the first aim of this study was to provide an overview of current load monitoring practices by surveying a broad sample of European elite football clubs. The second aim was to examine how these practices are impacted by the club belief on the benefit of EBP and the club financial resources. This information may provide more insight into the existing differences in implementation between elite clubs.

## Materials and Methods

### Participants

A closed online survey (Qualtrics, Provo, UT) was distributed from January to September 2019. Only senior clubs competing in the top league of the 25 highest-ranked and the second league of the 10 highest-ranked European federations were invited (UEFA country coefficient 2018/2019). This led to 197 invited clubs. All invitations were e-mailed to a member of the sports-science or sports-medicine department. Staff members were identified in collaboration with a contact person that was familiar with the national federation (*n* = 104), the authors own network (*n* = 32), and an additional search on LinkedIN (*n* = 61). A first reminder was sent if there was no response within 1 month. A maximum of three reminders was sent if the survey remained unanswered. Before commencement, an informed consent document was provided including information about the purpose and procedures of the survey, confidentiality, and anonymity. Participation was voluntary and no incentives were provided. The study was conducted according to the requirements of the Declaration of Helsinki and was part of a research project that was approved by the KU Leuven ethics committee (s57732).

### Design

The content of the survey was developed from an evidence- and practice-based perspective and was evaluated by the authors to make sure it was adequate for the purpose of the study. Next, the face validity of the survey was assessed by four applied researchers/practitioners with specific expertise in the field of load monitoring (BSc. degree (*n* = 1), PhD. degree (*n* = 2), Prof. degree (*n* = 1); > 10 years of experience in physical training), and one expert researcher in survey design and construction (Prof. degree). This resulted in four modified questions, one added question and seven deleted questions. The final version was pilot tested for usability by two staff members working in an elite youth academy (MSc. degree (*n* = 2); ± 3 years of experience in physical training). The survey was originally developed in English and translated by researchers into their native language (Dutch, French, German, Italian and Spanish). The researchers were familiar with the terminology used in the field of load monitoring. However, no cross-cultural validation was performed, which can be considered a limitation of the translation process. Back buttons were provided to allow participants to change their answers. Following each question, participants were allowed to provide extra information in a text box.

The survey consisted of 38 closed ended questions using a Likert scale or multiple choice (detailed information can be found in the Supplementary Material). In each question, staff members could indicate “no idea” if they were unable to answer the question correctly. All Likert scales contained five points and were fully labeled (1 = strongly disagree; 2 = somewhat disagree; 3 = neither agree nor disagree; 4 = somewhat agree; 5 = strongly agree). The implementation of load monitoring practices was examined by asking questions related to the types of data that are collected and the collection purposes (13 multiple choice questions), the applied analysis methods (seven Likert scale questions) and the involvement of staff members in the monitoring process (13 multiple choice question). The club belief on the benefit of EBP was surveyed via five Likert scale questions. The club financial resources were estimated via Transfermarkt (transfermarkt.com). This website uses crowd-based estimations to determine the market value of the club, consisting of the sum of individual player market values. Despite its limitations, Transfermarkt has proven to be accurate and reliable in estimating market values (Müller et al., 2017).

### Statistical Analyses

**Figure 1:**
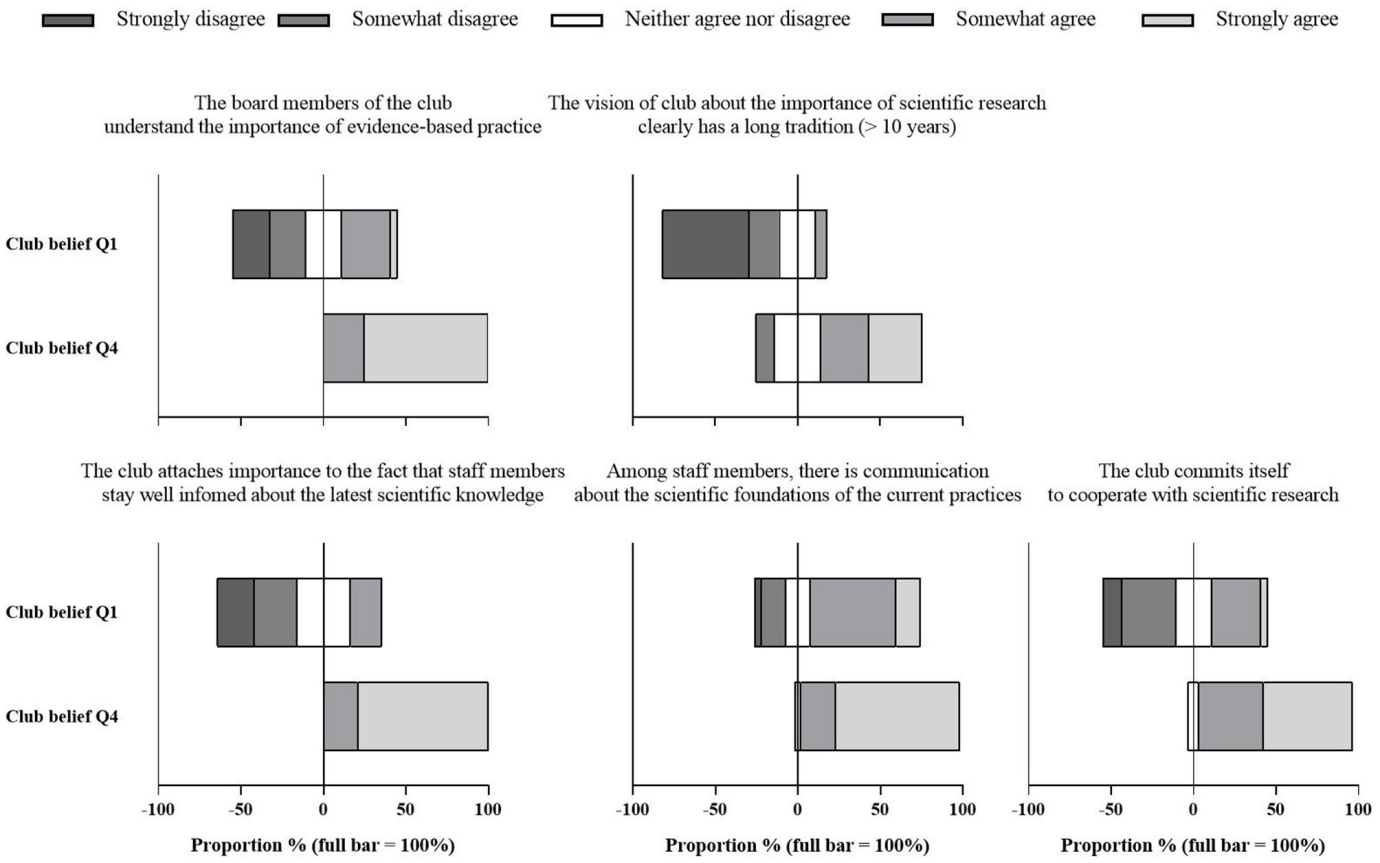
Responses to the five Likert scale questions examining the club belief on the benefit of EBP. Results are presented as the proportion of clubs in quartile 1 (Q1) and quartile 4 (Q4).

Data were exported into Microsoft Excel (Version 2016, Microsoft Corporation, Washington USA) and SPSS (version 26, IBM, New York, USA). Both the multiple choice and Likert scale questions were analyzed using frequency analysis and the results were presented and compared as the proportion of responded clubs, with single staff members representing their club. Clubs were categorized into quartiles for club belief and financial resources based on even distributions to examine how monitoring practices are impacted by both factors. Results were compared between quartile 1 (Q1) and quartile 4 (Q4) to ensure clear differences between groups. While the club market value was used to categorize clubs based on the club financial resources, the average response to five Likert scale questions was used to categorize clubs based on the club belief on the benefit of EBP. Although Likert scale responses are ideally treated as ordinal-level data, this approach was appropriate to distinguish clubs with different characteristics in terms of the club belief on the benefit of EBP (Figure 1). The internal consistency between the five questions was checked and found to be appropriate (Cronbach's Alpha = 0.74, mean inter-item correlation = 0.37).

## Results

The survey was completed by 99 representatives of clubs belonging to 15 federations. Clubs competed in the top league (*n* = 75) and the second league (*n* = 24) of their federation (response rate = 50%) (Table 1). Clubs were represented by sports-science (*n* = 82) and sports-medicine staff members (*n* = 17), having on average 9.21 ± 5.48 and 10.47 ± 9.08 years of experience in football and 5.09 ± 4.23 and 8.35 ± 9.65 years of experience in their current club, respectively. Staff members were identified in collaboration with a contact person that was familiar with the national federation (*n* = 61; response rate = 58%), the authors own network (*n* = 21; 62%) and an additional search on LinkedIN (*n* = 17; 27%).

**Table 1:** Number of clubs invited per federation^a^ and corresponding response rates.

	**Response rate**			**Belief on the benefit of EBP**		**Financial resources**	
	**Invited** **(#)**	**Responded** **(#)**	**Rate** **(%)**	**Q1** **(%)**	**Q4** **(%)**	**Q1** **(%)**	**Q4** **(%)**
SPA	21	12	57	33	42	8	50
ENG	18	8	44	13	75	0	75
ITA	12	5	42	0	0	20	40
GER	20	7	35	43	29	14	57
FRA	21	14	67	50	7	21	14
RUS	2	2	100	0	50	0	50
POR	20	12	60	42	17	42	25
BEL	24	13	54	0	46	23	0
TUR	1	0	0	/	/	/	/
NED	11	8	64	25	25	13	13
AUT	6	3	50	67	0	67	0
CZE	1	1	100	0	100	0	0
GRC	2	1	50	0	100	0	0
CRO	2	0	0	/	/	/	/
DNK	12	5	42	40	20	60	0
CHE	3	0	0	/	/	/	/
CYP	1	0	0	/	/	/	/
SRB	2	0	0	/	/	/	/
SCO	9	3	33	0	0	67	0
SWE	5	5	100	20	0	60	0
NOR	2	0	0	/	/	/	/
POL	2	0	0	/	/	/	/
	**197**	**99**	**50**				

a *Ordered by the UEFA country coefficient of the 2018/2019 season (Spain = highest ranked, Poland = lowest ranked)*.

*Percentage of clubs allocated to quartile 1 (Q1) and quartile 4 (Q4) for club belief on the benefit of evidence-based practice (EBP) and club financial resources per national federation*.

### Overview of Load Monitoring Practices

Table 2 shows the proportion of clubs that collect external load, internal load and training outcome data for the different collection purposes. Proportions were high for all types of data but were higher for external load data (65–97%) compared to internal load (47–90%) and training outcome data (53–94%). Almost all respondents indicated to collect data for training planning, performance assessment and rehabilitation purposes. More than 50% also indicated to collect data for game management and youth development.

**Table 2:** Proportion of clubs (%) that collect external load, internal load and training outcome data for the different purposes of load monitoring.

	**Total**	**Belief on the benefit of EBP**		**Financial resources**	
	**%**	**Q1 (%)**	**Q4 (%)**	**Q1 (%)**	**Q4 (%)**
**Training Planning**					
External load	97	93	100	88	100
Internal load	90	78	100	80	100
Training outcome	94	85	100	84	100
**Performance Assessment**					
External load	96	89	100	84	100
Internal load	69	67	79	56	80
Training outcome	80	78	82	80	88
**Rehabilitation**					
External load	91	89	96	83	96
Internal load	89	85	96	83	92
Training outcome	87	81	93	96	96
**Game Management**					
External load	65	56	70	48	71
Internal load	47	40	52	35	50
Training outcome	53	52	56	48	63
**Youth Development**					
External load	70	64	81	41	81
Internal load	58	48	69	36	62
Training outcome	57	52	62	50	57

*Proportions are presented for the total sample and for quartile 1 (Q1) and quartile 4 (Q4) for both club belief on the benefit of evidence-based practice (EBP) and club financial resources*.

**Figure 2:**
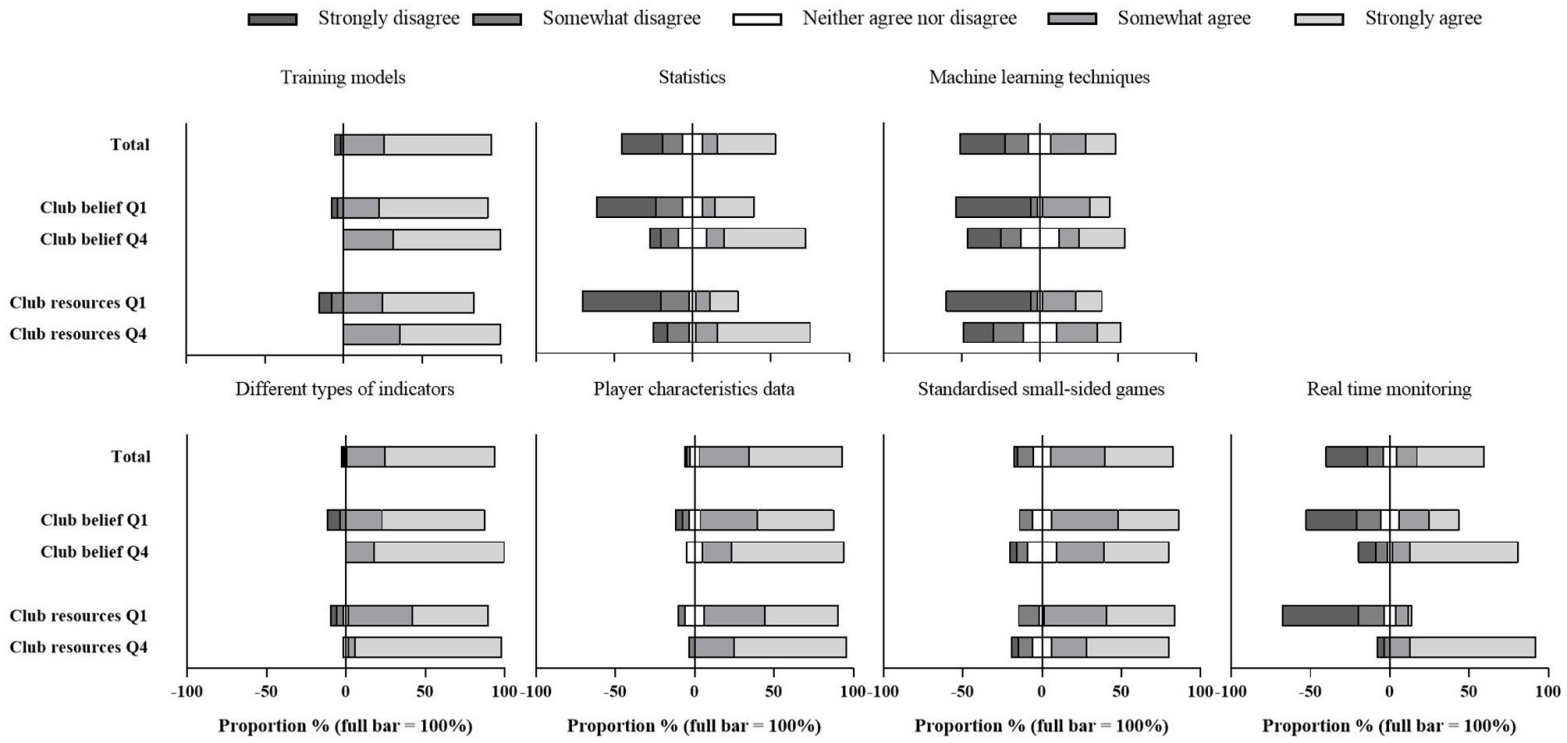
Responses to the seven Likert scale questions examining the analysis methods. Results are presented as the proportion of clubs for the total sample and for quartile 1 (Q1) and quartile 4 (Q4) for both club belief on the benefit of evidence-based practice (EBP) and club financial resources.

To analyse these data, respondents indicated to use training models such as the acute-chronic workload ratio, fitness-fatigue model, or the monotony/strain scores (Figure 2). In line with the extensive data collection presented in Table 2, respondents confirmed to combine different types of indicators to make accurate interpretations. In addition, respondents indicated to individualize the analysis based on the physical characteristics of the players and acknowledged to use standardized small-sided games for analysis purposes. Responses were less univocal regarding the use of statistics and machine learning techniques to detect relevant relations in the data and for the use of real-time monitoring during training and matches.

Table 3 shows the involvement of the different types of staff members in the monitoring process. Coaching staff members were most involved in the final phases of the process (i.e., discussion and application), with the head and assistant coach being more involved than the goalkeeper coach. Sports-science staff members were involved throughout the entire process. Sports-medicine staff members were most involved in the discussion phase. External staff member presence was somewhat unusual. If present, external staff members' involvement depended on the type of external staff member.

**Table 3:** Presence and involvement of the different type of staff members in the different phases of the monitoring process.

	**Presence**	**Involvement if present**				
	**(%)**	**Collection** **(%)**	**Analysis** **(%)**	**Reporting** **(%)**	**Discussion** **(%)**	**Application** **(%)**
**Coaching Staff**						
Head coach	100	4	34	18	77	74
Assistant coach	100	11	40	18	69	64
Goalkeeper coach	100	11	29	13	46	39
**Sports-Science Staff**						
Fitness or S&C coach	99	82	85	82	96	84
Performance manager	42	22	26	26	38	26
Sport scientist	43	40	43	42	39	31
**Sports-Medicine Staff**						
Physiotherapist	99	25	34	25	60	28
Medical manager	72	18	25	23	45	21
Club doctor	97	20	30	22	61	20
**External Staff**						
University researcher	39	18	22	9	14	3
Internship students	44	27	16	15	8	1
Athlete management company	14	7	14	12	5	3
Self-employed consultant	23	6	11	8	10	4

*Values are presented as the proportion of clubs (%)*.

### Impact of Club Belief on the Benefit of EBP and Impact of Club Financial Resources

**Figure 3:**
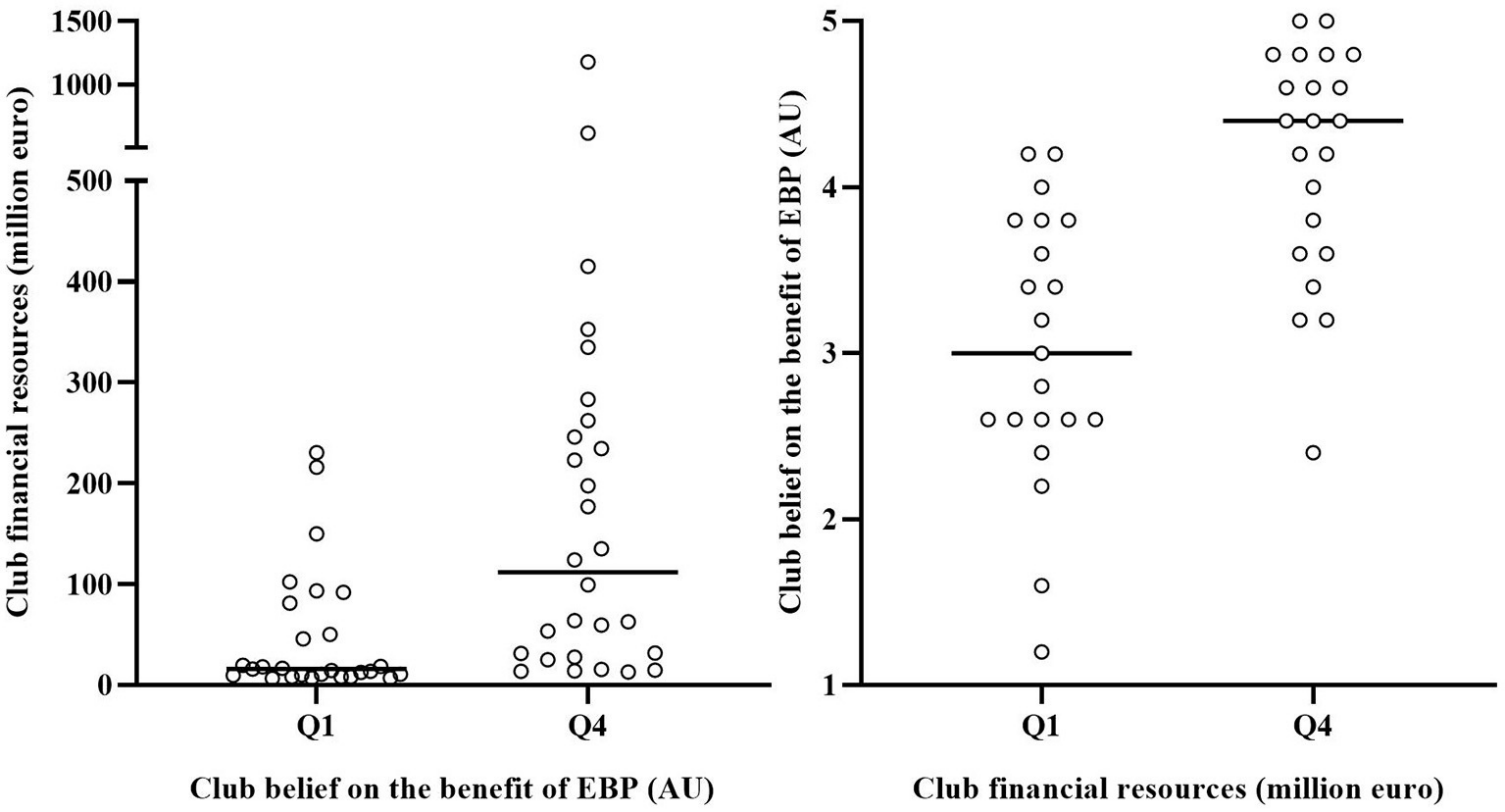
Relationship between the club belief on the benefit of evidence-based practice (EBP) and the club financial resources. Lines represent median values.

The average response to the five Likert-scale questions (five-point) examining the club belief on the benefit of EBP was 3.64 ± 0.80 (Q1 = range: 1.20–3.20; X: 2.69 ± 0.53) (Q4 = range: 4.20–5.00; X: 4.51 ± 0.29). The average club financial resources, expressed as the club market value, was 102.02 ± 164.56 million euro (Q1 = range: 2.30–13.88; X: 9.22 ± 3.08) (Q4 = range: 124.30–1180.00; X: 312.03 ± 213.81). Table 1 shows that club financial resources are greater in high-level federations compared to lower level federations. No clear relation between the federation level and the average club belief on the benefit of EBP was observed. However, Figure 3 shows that clubs with high resources tend to have a stronger belief in the benefit of EBP and vice versa.

Both factors clearly contribute to a more diverse data collection across the different load monitoring purposes (Table 2). This is indicated by a higher proportion of clubs collecting the different types of data in Q4 compared to Q1. Regarding analysis methods, Figure 2 shows that Likert scale responses were more positive when club belief was stronger and resources were greater. However, clear differences were only observed for the use of statistics and real-time monitoring. Table 4 presents the impact of the club belief and resources on the presence and involvement of the different types of staff members in the monitoring process. The performance manager, sport scientist, medical manager and external staff members were clearly more present in Q4 compared to Q1 for both club belief and resources. University researchers, athlete management companies and self-employed consultants were more involved (if present) in Q1 compared to Q4.

**Table 4:** Presence (and involvement) of the different types of staff members in the monitoring process.

**Presence** **(Involvement if present)**	**Belief on the benefit of EBP**		**Financial resources**	
	**Q1**	**Q4**	**Q1**	**Q4**
	**%**	**%**	**%**	**%**
**Coaching Staff**				
Head coach	100 (79)	100 (96)	100 (96)	100 (91)
Assistant coach	100 (76)	100 (96)	100 (86)	100 (87)
Goalkeeper coach	100 (58)	100 (67)	100 (68)	100 (68)
**Sports-Science Staff**				
Fitness or S&C coach	100 (100)	100 (100)	96 (96)	100 (100)
Performance manager	23 (83)	36 (94)	37 (78)	71 (94)
Sport scientist	30 (100)	71 (100)	21 (100)	76 (100)
**Sports-Medicine Staff**				
Physiotherapist	96 (73)	100 (86)	100 (76)	100 (83)
Medical manager	67 (83)	79 (82)	52 (69)	96 (74)
Club doctor	92 (71)	100 (79)	96 (59)	96 (83)
**External Staff**				
University researcher	19 (100)	57 (69)	24 (83)	44 (73)
Internship students	37 (46)	80 (62)	21 (60)	48 (67)
Athlete management company	24 (96)	32 (76)	29 (71)	39 (33)
Self-employed consultant	11 (100)	36 (50)	8 (100)	44 (77)

*Values are presented as the proportion of clubs (%) in quartile 1 (Q1) and quartile 4 (Q4) for both the club belief on the benefit of evidence-based practice (EBP) and the club financial resources*.

## Discussion

The primary aim of this study was to provide an overview of the load monitoring practices in European elite football. A secondary aim was to better understand the differences in practices between clubs by examining the impact of the club belief on the benefit of EBP and the club financial resources. The main findings related to the first aim are that data are collected extensively but monitoring external load still receives more attention than monitoring internal load and training outcomes. Data collection and analysis were shown to be largely performed by sports-science staff members. Coaching and sports-medicine staff members were involved in data discussion to ensure shared decision-making. With respect to the second aim, the main finding is that a stronger club belief and greater financial resources facilitate a more extensive data collection and analysis and stimulate the involvement of specialist staff members such as the sport scientist. The influence of response bias should be taken into account when interpreting the results of this study. It is likely that the response rate (50%) is biased toward scientifically interested staff members. This could have resulted in an overestimation of the reported practices. In addition, practices may be overestimated because the survey queried for perceived rather than actual practice.

### Overview of Load Monitoring Practices

The majority of respondents indicated that their club collect data in an extensive way, measuring external load, internal load and training outcome data for a multitude of purposes. It was observed that the monitoring of external load received more attention than the monitoring of internal load, which confirms the findings from previous studies (Akenhead and Nassis, 2016; Weston, 2018). This can be explained by the better usability of external load data for training planning and the current challenges of monitoring internal load (Vanrenterghem et al., 2017; Verheul et al., 2020). However, it has been stated that the combined monitoring of external and internal load is necessary to better understand the relation between load and training outcomes (Impellizzeri et al., 2019). While recent research in the field of load monitoring indeed argued for a multifactorial approach, the collection of different types of variables increases the amount of available data, thereby making it challenging to use the data effectively (Weaving et al., 2017). When the data is only collected and reported (descriptive level), the added value of monitoring may be rather low. By analyzing the relation between the different types of data (diagnostic level), staff members can develop insights that better support decision-making and consequently increase the value of monitoring (Ward et al., 2018; Thornton et al., 2019).

In the current study, questions were asked to gain a better understanding about the level of load monitoring analysis applied in the clubs. In line with the extensive data collection, the majority of respondents agreed to make interpretations based on different types of indicators. Around 50% of the respondents reported to use statistics to detect relations in the data, which can be considered an important step in the diagnostic level of analysis. In addition, around 40% of the respondents agreed to use machine learning techniques for this purpose. This result is somewhat surprising given the lack of guidelines to use such methods effectively and the general difficulty of applying machine learning (Claudino et al., 2019). So, although data is collected extensively, the current study shows a considerable diversity in data analysis which suggests that analysis from the gathered data is often limited to reporting against what has been gathered previously within the club. To progress the field from descriptive toward diagnostic analysis, a more consistent implementation of analysis methods that focus on the relations between different types of data is still needed.

This study provides evidence about the involvement of the different staff members in the monitoring process. Similar to other business organizations, well-defined staff responsibilities may create effective communication and shared decision-making, thereby increasing the value of load monitoring in general. The results showed that sports-science staff members were most involved throughout the entire process. This indicates that sports-science staff members carry the main responsibility for load monitoring. Coaching staff members were mainly involved in data discussion and application. Remarkably, the goalkeeper coach was clearly less involved than the head and assistant coach. This may be due to differences in the desired performance outcomes, injury risks and corresponding training methods (Moreno-Pérez et al., 2020). In fact, despite recent improvements, it remains difficult to quantify the external and internal load of a goalkeeper objectively (Malone et al., 2018). Similar to coaching staff members, sports-medicine staff members were mainly involved in data discussion which indicates that decision-making is shared among staff-members. Because the responsibilities regarding data collection and analysis belong in particular to sports-science staff members, effective communication to the other staff members is of utmost importance to improve decision-making (Ekstrand et al., 2019).

The involvement of external staff members was also examined. In general, their presence was somewhat unusual. If present, internship students were most involved in data collection and analysis. Although these tasks contain challenging learning opportunities, students are possibly involved here to perform the more repetitive tasks (Malone, 2017). This may limit the opportunity to learn, especially with regard to decision-making in which they are clearly less involved. Similarly, University researchers such as (embedded) PhD students were mainly involved in data collection, indicating the need for assistance in collecting huge amounts of data. Given the (expected) familiarity of researchers with statistics, clubs may consider the benefit of involving them when progressing from descriptive to diagnostic analysis.

### Impact of Club Belief on the Benefit of EBP and Impact of Club Financial Resources

The second aim of this study was to examine the impact of the club belief on the benefit of EBP and the impact of the club financial resources on the monitoring practices in European elite football. It was observed that a stronger belief and greater resources facilitate an extensive data collection. Increased resources may stimulate the purchase of measurement technology. A strong belief on the benefit of EBP may further promote data collection practices by creating a culture that values the use of data. Both factors may also stimulate the use of in-depth analysis methods. Diagnostic analysis requires time, knowledge and data skills and may therefore be facilitated by a stronger belief and greater resources. The results indeed showed that the respondents representing clubs with a stronger belief and greater resources agreed more to the question if they use statistics to detect relations in the data. This may be related to the observation that these factors stimulate the employment of specialist staff members such as the sport scientist. In these clubs, specialist staff members may have an important impact on the efficiency and consistency of the data collection, the depth of the analysis and the procedures to report and communicate the data, thereby providing more time for other staff members to improve their training and therapy practices (McCall et al., 2016). In smaller clubs, this range of tasks may be largely performed by single staff members, which possibly limits the quality and impact of load monitoring. So, an organizational culture that is characterized by a strong belief on the benefit of EBP may facilitate the implementation of load monitoring practices. This indicates the importance of staff, player, and director commitment to load monitoring. The actual potential of monitoring practices may, however, still be largely determined by the financial resources of the clubs and their corresponding capacity to purchase technology and employ specialist staff members. Whilst these high-level clubs may in the first place wish to protect their competitive advantage by protecting their methods, one could argue that from the perspective of a duty of care for all, they would be best placed to play a leading role in generating and sharing knowledge to improve training practices and player health (Ramirez-Lopez et al., 2020).

### Strengths, Limitations and Future Directions

The current study provides a comprehensive overview of the load monitoring practices applied by European elite football clubs. Data was collected between January and September 2019. Because practices within elite football are highly dynamic, the results of this study must be interpreted in the context of this time period. Information was included about both the used methods and the staff involvement. Furthermore, this study provides insight into the existing differences in implementation between clubs by examining the implementation context. Although previous studies suggested the club belief on the benefit of EBP and the club financial resources as influencing factors, this study is to first to examine the actual impact of both factors on the monitoring practices. In comparison with previous studies, this examination included a larger and more representative sample of elite football clubs (Akenhead and Nassis, 2016; Weston, 2018). We acknowledge, however, that response bias can never be excluded because scientifically interested staff members will likely have been more willing to contribute to the study. As this survey queried for perceived rather than actual practice, their judgements may at the same time be prone to an overestimation of their actual performance of what is a difficult task (Moore and Schatz, 2017). Response collection may also be too narrow because clubs were represented by a single sports-science or sports-medicine staff member. Although previous studies already attempted to examine differences in perceptions between staff members, an investigation from different perspectives may be required by combining coaching, sports-science, sports-medicine and external staff members' perceptions (Weston, 2018; Malone et al., 2019). Even more important could be the examination of other club members' perceptions, such as the board members of the club, whose directives influence monitoring practices financially and culturally, and the players, who are the end-customers of the monitoring process (Fullagar et al., 2019; Gouttebarge et al., 2019). In contrast to widening the responses, we believe that future research involving qualitative methods such as 1-on-1 interviews and focus groups may provide valuable in-depth insights into development needs concerning the practical implementation of monitoring practices (Harper and McCunn, 2017). While this study provided evidence for the impact of the club beliefs on the benefit of EBP and the club financial resources on the implementation of monitoring practices, more in-depth analyses may provide greater insight into the underlying mechanisms between these club characteristics and the implementation of monitoring practices. Such analyses ideally result in the development of specific guidelines for clubs to improve their monitoring process based on their implementation context (i.e., organizational culture and resources).

## Conclusion

This study first aimed to provide an overview of load monitoring practices in European elite football. These practices are currently characterized by an extensive data collection, including external load, internal load and training outcome data. However, the results demonstrate that external load data still receive more attention than internal load and training outcome data. Considerable diversity in data analysis was observed indicating that analysis is often restricted to the descriptive level, limiting the development of insights in the diagnostic level, which is important to improve data-informed decision-making. The results showed that decision-making is shared among staff members but data collection and analysis belong in particular to sports-science staff members. A secondary aim of the study was to examine the impact of the club belief on the benefit of EBP and the impact of the club financial resources on these practices. The results showed that both a stronger belief and greater financial resources facilitate the extensive collection of data, the use of diagnostic analysis techniques and the employment or collaboration with specialist staff members. The influence of response bias should be taken into account when interpreting the results of this study.

## Data Availability Statement

The raw data supporting the conclusions of this article will be made available by the authors, without undue reservation.

## Ethics Statement

The studies involving human participants were reviewed and approved by KU Leuven ethics committee (s57732 file number). The patients/participants provided their written informed consent to participate in this study.

## Author Contributions

KH, JV, AJ, LR, MB, and WH contributed to the design and implementation of the research, to the analysis of the results, and to the writing of the manuscript. All authors contributed to the article and approved the submitted version.

## Conflict of Interest

The authors declare that the research was conducted in the absence of any commercial or financial relationships that could be construed as a potential conflict of interest.
