# Supplementary material for: Load Monitoring Practice in European Elite Football and the Impact of Club Culture and Financial Resources
Source: Front Sports Act Living. 2021 May 20;3:679824. doi: 10.3389/fspor.2021.679824 (PMC8173105; doi:10.3389/fspor.2021.679824)
Supplement: Supplementary file 1 [file Data_Sheet_1.docx]

Supplementary Material – Survey design

# Personal information

- Club
- Job Title
  - Sports-medicine staff member
  - Sports-science staff members
- Experience (years)
  - In football
  - In current club

# Types of data collected and collection purposes (multiple-choice grid)

- Question
  - Please select the objectives and interventions for which you analyse player data using the following variables. You can select more than one variable per objective and intervention.
- Variables
  - External load (e.g. global navigation satellite system, local position measurement system, accelerometer)
  - Internal load (e.g. heart rate, lactate, rating of perceived exertion)
  - Training outcome (e.g. injury, fitness, neuromuscular fatigue, well-being)
- Objectives and interventions
  - Training planning
    - General planning (micro-, meso- and macrocycle)
    - Specific planning (passing drill, small-sided games, running drill)
    - Individual adjustment (overuse, detraining, underperformance)
    - Individual adjustment (physical development)
  - Performance assessment (team level)
    - Determination (evolution) team performance
    - Determination (evolution) player performance
    - Comparison performance between players
  - Game management
    - Selection starters, non-starters and reserves
    - Planning substitution strategy during the game
  - Rehabilitation
    - Planning return to sport process
    - Determination return to group training during rehabilitation
    - Determination return to competition during rehabilitation
  - Youth development
    - Physical preparation youth players for professional football demands

# Applied analysis methods (Likert scale)

- Question
  - Please answer to the following statements
- Statements
  - Do you use
    - Training models (e.g. acute-chronic workload ratio, fitness-fatigue model, monotony)
    - Statistics (e.g. Z-scores, analysis of variance, magnitude-based inferences)
    - Machine learning techniques
    - Different types of indicators to make accurate interpretations
    - Player characteristics data to individualise the analysis
    - Standardised small-sided games
    - Real time-monitoring

# Staff involvement (multiple choice grid)

- Question
  - Please select which role staff members/organisations have in the analysis process. You can select more than one role per staff member/organisation.
- Roles
  - Not present
  - No role
  - Data collection
  - Data analysis
  - Data reporting
  - Data discussion
  - Data application
- Staff members/organisations
  - Coaching department
    - Head coach
    - Assistant coach
    - Goalkeeper coach
  - Sports-science department
    - Fitness or S&C coach
    - Performance manager
    - Sport scientist
  - Sports-medicine department
    - Physiotherapist
    - Medical manager
    - Club doctor
  - External staff members
    - University researchers
    - Internship students
    - Athlete management company
    - Self-employed scientific consultant

# Club belief on the benefit of evidence-based practice (Likert scale)

- Question
  - Please answer to the following statements
- Statements
  - The board members of the club understand the importance of evidence-based practice.
  - The vision of the club about the importance of scientific research clearly has a long tradition (> 10 years).
  - The club attaches importance to the fact that staff members stay well informed about the latest scientific knowledge.
  - Among staff members, there is communication about the scientific foundations of the current practices.
  - The club commits itself to cooperate with scientific research.
